# *Echinacea purpurea* extract intervention for counteracting neurochemical and behavioral changes induced by bifenthrin

**DOI:** 10.1007/s11011-023-01303-6

**Published:** 2023-12-27

**Authors:** Khaled G. Abdel-Wahhab, Rehab S. Sayed, Doaa G. EL-Sahra, Laila K. Hassan, Ghada M. Elqattan, Fathia A. Mannaa

**Affiliations:** 1 Medical Physiology Department, National Research Centre, Giza, 12622 Egypt; 2 Regional Center for Food and Feed, Agriculture Research Centre, Giza, Egypt; 3 Modern University for Technology and Information, Cairo, Egypt; 4 Dairy Department, National Research Centre, Giza, 12622 Egypt

**Keywords:** Herbal extract, Neurotoxicity, Oxidative stress, Brain, Behavior, Rats

## Abstract

This study was conducted to elucidate the possible protective efficiency of *Echinacea purpurea* hydroethanolic extract (EchEE) against bifenthrin (BIF)-induced neuro-chemical and behavioral changes in rats. Total phenolics content, reducing power and radical scavenging activity of EchEE were estimated. Four groups of adult male albino rats were used (10 rats each) as follows: 1) Control healthy rats ingested with placebo, 2) Healthy rats orally received EchEE (465 mg/kg/day), 3) Rats intoxicated with BIF (7mg/kg/day) dissolved in olive oil, and 4) Rats co-treated with EchEE (465 mg/kg/day) besides to BIF (7mg/kg/day) intoxication. After 30 days, some neuro-chemical and behavioral tests were assessed. The behavioral tests revealed that rats received BIF exhibited exploratory behavior and spatial learning impairments, memory and locomotion dysfunction, and enhanced anxiety level. Biochemical findings revealed that BIF induced-oxidative stress in the cortex and hippocampus; this was appeared from the significant rise in malondialdehyde (MDA) and nitric oxide (NO) levels, coupled with decreased catalase (CAT), superoxide dismutase (SOD), paraoxonase-1 (PON-1) activities, and reduced glutathione (GSH) level in both brain areas. Also, BIF induced a significant increase caspas-3, tumor necrosis factor alpha (TNF), and interleukin-1beta (IL-1ß) in both areas; dopamine and serotonin levels, and ACh-ase activity were markedly decreased in both areas. Interestingly, treatment of rats with EchEE in combination with BIF resulted in a significant decrease in oxidative stress damage, and modulation of the apoptotic and pro-inflammatory markers. Also, EchEE markedly improved behavioral activities and neurotransmitters level that were impaired by BIF. In conclusion, the present study clearly indicated that EchEE can attenuate brain dysfunction induced by pesticides exposure through preventing the oxidative stress. This may be attributed to its high antioxidant component.

## Introduction

Pyrethrinoids are synthetic pesticides that are widely used in protection of crops from insects, weeds, fungi, and molds (Beghoul et al. 2017). Chronic exposure to pyrethroids via dust, indoor air, and/or consumption of contaminated food products can have harmful effects on human health (Nieradko-Iwanicka et al. 2015; Beghoul et al. 2017). Pyrethroids are neurotoxic substances, and act on the voltage-gated sodium, calcium, and chloride channels of the central nervous system in mammals (Soderlund et al. 2002; Soderlund 2012).

Bifenthrin (BIF) has a potent insecticidal activity; it is greatly used in orchards, nurseries, and homes (Syed et al. 2016). It belongs to the type I pyrethroids that lacks the alpha-cyano group and induces aggressive sparring, tremors increment, and prostration in animals (Scollon et al. 2011; Syed et al. 2018). Accumulating studies have indicated that BIF exposure induces neurodegenerative effects in various animal species like motor incoordination, deficits in motor activity and cognitive impairment (Gargouri et al. 2018a; Syed et al. 2018; Gomaa et al. 2021); however, it has been classified by the WHO as class II moderately hazardous pesticide (Syed et al. 2018).

Oxidative stress is a common mechanism of toxicity associated with many pesticides (Lukaszewicz-Hussain 2008); whereas exposure to pyrethroids is known to induce production of free radicals and reactive oxygen species (ROS) that lead to a damaging effect on animal organs (Syed et al. 2018; Gomaa et al. 2021); therefore, natural products containing antioxidants such as phenolic compounds, flavonoids, tocopherol, alkamides, and saponins, may exhibit protective effects against the toxic action of pesticides.

*Echinacea purpurea*, known as Purple Coneflower; this herbal plant belonging to *Asteraceae* family, and has received a great interest for its modulatory ability of different inflammatory disease such as skin inflammation, sore throat, and swollen gums (Kindscher 1989). Also, the extracts from *Echinacea purpurea* leaves shown potent pharmacological activities such as antibacterial, antifungal, antiviral (Hudson 2012), immunomodulatory (Hudson 2012; Manayi et al. 2015), anti-inflammatory (Borchers et al. 2000), and antioxidant effects (Chen et al. 2015; Aarland et al. 2017). *Echinacea Purpurea* preparations are also used to improve hyperglycemia and insulin resistance (Mao et al. 2021), and regulate hyperthyroid hormones (EL-Sahra et al. 2022). Similarly, *Echinacea purpurea* ethanolic extract (EchEE) was found to increase sperm motility, protect sperm morphology, and improve mitochondrial membrane potential (Mao et al. 2021). Caffeic acid derivatives, alkamides, and polysaccharides are the main components, of EchEE, that are responsible for its medicinal properties (Bone 2004). Additionally, several studies have demonstrated the safety and nontoxic effects of the *Echinacea* preparations at the recommended doses (Xu et al. 2021); therefore, the current study was designed to estimate the attenuating efficiency of the hydroethanolic extract of *Echinacea purpurea* flowers against BIF-induced neurochemical and behavioral changes in rats.

## Materials and methods

### Plant and ethanolic extraction

Flowers of* Echinacea purpurea* plant were bought from a local supplier, and identified by a special botanist and the plant was found carrying the taxonomic serial number 3728. The grinded flowers were immersed in 70% ethanol (1:5 w/v) for 7 days; then, the mixture was filtered through Whatman 1 filter paper, and the solvent was then removed using a rotatory evaporator (Hei-VAP Rotary Evaporator, Germany); while the moisture residue was removed using a freeze drier (Snijders Scientific-tilburg, Holland) under pressure, 0.1 to 0.5 mbar and temperature -35 to -41°C conditions (Todd et al. 2015). The resultant dry extract was stored at -20 °C until in vitro and in vivo assessments.

### In vitro assessments

Spectrophotometrically (Agilent Cary 100 UV–Vis spectrophotometer, Santa Clara, California, USA), 2,2-diphenyl-1-picrylhydrazyl (DPPH) scavenging activity of the extract was determined following the method described by Nogala-Kalucka et al. (2005), and radical scavenging activity (RSA) was calculated using the equation $\left[\mathrm{RSA\%}= \frac{{\varvec{A}}{\varvec{b}}{\varvec{s}}{\varvec{o}}{\varvec{r}}{\varvec{b}}{\varvec{a}}{\varvec{n}}{\varvec{c}}{\varvec{e}}\boldsymbol{ }{\varvec{o}}{\varvec{f}}\boldsymbol{ }{\varvec{c}}{\varvec{o}}{\varvec{n}}{\varvec{t}}{\varvec{r}}{\varvec{o}}{\varvec{l}}-{\varvec{A}}{\varvec{b}}{\varvec{s}}{\varvec{o}}{\varvec{r}}{\varvec{b}}{\varvec{a}}{\varvec{n}}{\varvec{c}}{\varvec{e}}\boldsymbol{ }{\varvec{o}}{\varvec{f}}\boldsymbol{ }{\varvec{s}}{\varvec{a}}{\varvec{m}}{\varvec{p}}{\varvec{l}}{\varvec{e}}}{{\varvec{A}}{\varvec{b}}{\varvec{s}}{\varvec{o}}{\varvec{r}}{\varvec{b}}{\varvec{a}}{\varvec{n}}{\varvec{c}}{\varvec{e}}\boldsymbol{ }{\varvec{o}}{\varvec{f}}\boldsymbol{ }{\varvec{c}}{\varvec{o}}{\varvec{n}}{\varvec{t}}{\varvec{r}}{\varvec{o}}{\varvec{l}}} \times 100\right]$

Total phenolics content (TPC) in the extract was estimated as catechin equivalent using catechin standard curve as described by Jayaprakasha et al. (2003).

Finally, ferric-reducing power of the extract was estimated as ascorbic acid equivalent from the standard curve of ascorbic acid as described by Sethiya et al. (2014).

### HPLC analysis of phenolic ingredient

HPLC analysis of the extract was carried out using an Agilent 1260 series. The separation was carried out using Eclipse C18 column (4.6 mm × 250 mm i.d., 5 μm). The mobile phase consisted of water (A) and 0.05% trifluoroacetic acid in acetonitrile (B) at a flow rate 0.9 ml/min. The mobile phase was programmed consecutively in a linear gradient as follows: 0 min (82% A); 0–5 min (80% A); 5–8 min (60% A); 8–12 min (60% A); 12–15 min (82% A); 15–16 min (82% A) and 16–20 (82%A). The multi-wavelength detector was monitored at 280 nm. The injection volume was 5 μl for each of the sample solutions. The column temperature was maintained at 40 °C.

### Animals and treatments

Adult male albino rats weighing 150-200g were obtained from the Animal House Colony of the National Research Centre, Egypt. Excess of rodent pellets diets and tab water were always available. All animals received human care in compliance with the standard institutional criteria for the care and use of experimental animals according to standard guidelines. After being acclimatized, the animals were divided into four groups (10 rats each) as follows: 1) healthy control rats ingested olive oil, 2) healthy rats ingested EchEE (465 mg/kg/day) dissolved in distill water (Mao et al. 2021), 3) healthy rats orally intoxicated with bifenthrin (BIF) dissolved in olive oil (7mg/kg/day) (Syed et al 2018), and 4) healthy rats co-treated with EchEE (465 mg/kg/day) besides to BIF (7mg/kg/day) intoxication. The experiment lasted for 30 days, and then all animals were subjected to the following behavioral tests.

### Open field test

In this test, each animal was gently placed into a corner of the cleaned and sterilized planed-arena and observed for 3 min; exploratory behaviors (ambulation and crossing of squares as well as rearing) were scored as absolute count (Gould et al. 2009).

### Y-Maze test

The short-term memory and locomotor activity was conducted according to Wright et al. (2006); the rats were video tracked through 5 min using video-tracking system (Anymaze 4.20, Stoelting, USA) that record the number of arm entries and distance travelled by each animal. Alternation percent was calculated according to the equation:

$$\mathrm{Alternation}\;\mathrm{percent}=\frac{total\;alterations}{entries\;number-2}\ast100$$

### Modified elevated plus maze test

Depending on the aversion of rats to the open space, the anxiety level was measured as described by Hlinák & Krejcí (2000).

### Novel object recognition test

Novel object recognition test (hippocampus dependent memory impairment) was measured via automated-tracking of rats using Video-tracking system (Anymaze 4.20, Stoelting, USA); all exploratory actions were measured as explained by Gümüş et al. (2018).

### Brain tissue sampling

Post last administration and assessment of behavioral tests, the animals were euthanized by sudden decapitation, and brain of each animal was excised and both cortex and hippocampus regions were anatomized; halve number of each group was ultrasonically homogenized (SONICS homogenizer, France) in phosphate buffer (0.1 M, pH 7.4) at ratio of 1:10 (w/v) and cool centrifuged (Hettich centrifuge, NEWTOWN CT, USA) at 5000 rpm for 10 min. The obtained supernatant was used for biochemical determinations, while the second halve (of each group) was homogenized in 0.1 M perchloric acid containing 3, 4-dihydroxybenzylamine at a final concentration of 25 ng/ml, for estimation of biogenic amines (dopamine and serotonin).

### Biochemical measurements

Spectrophotometrically, the end product of lipid peroxidation (malondialdehyde, MDA) was determined using the chemical method described by Ruiz-Larrea et al. (1994); also, the levels of nitric oxide (NO) and reduced glutathione (GSH) as well as the activities of catalase (CAT), and superoxide dismutase (SOD) were determined in both brain areas using reagent kits obtained from Biodiagnostic, Giza, Egypt. Paraoxonase-1 (PON-1) activity in the brain areas was carried out chemically according to the kinetic method of Eckerson et al. (1983).

Using ELISA (Dynatech Microplate Reader Model MR 5000, 478 Bay Street, Suite A213 Midland, ON, Canada), tumor necrosis factor alpha (TNF), interlukin-1 beta (IL-1β) and caspase-3 levels were measured in cortex and hippocampus using rats' reagent ELIZA-kits purchased from SinoGeneClon Biotech Co, Hang Zhou, China.

The activity of acetylcholinesterase (AchE-ase) was measured spectrophotometrically following the method of Ellman et al. (1961). Dopamine and serotonin levels were determined using high performance liquid chromatography (Agilent 1100 HPLC, USA), following the method described by Kim et al. (1987).

### Statistical analysis

The analysis of data was done using one way ANOVA followed by Duncan post hoc test at level of *p* ≥ *0.05* using SAS program software; copyright (c) 1998 by SAS Institute Inc., Cary, NC, USA.

## Results

### In vitro study

**Fig. 1:**
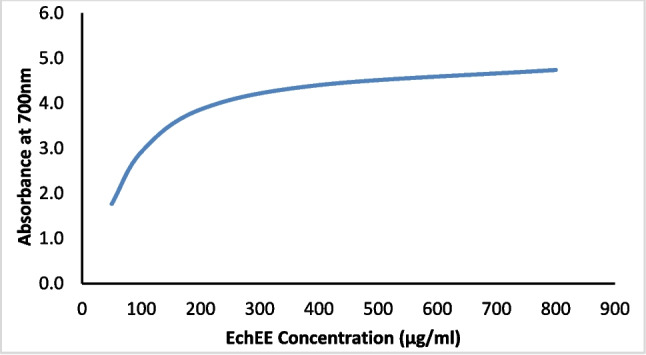
Reducing ability of *Echinacea purpurea* ethanolic extract (EchEE)

**Fig. 2:**
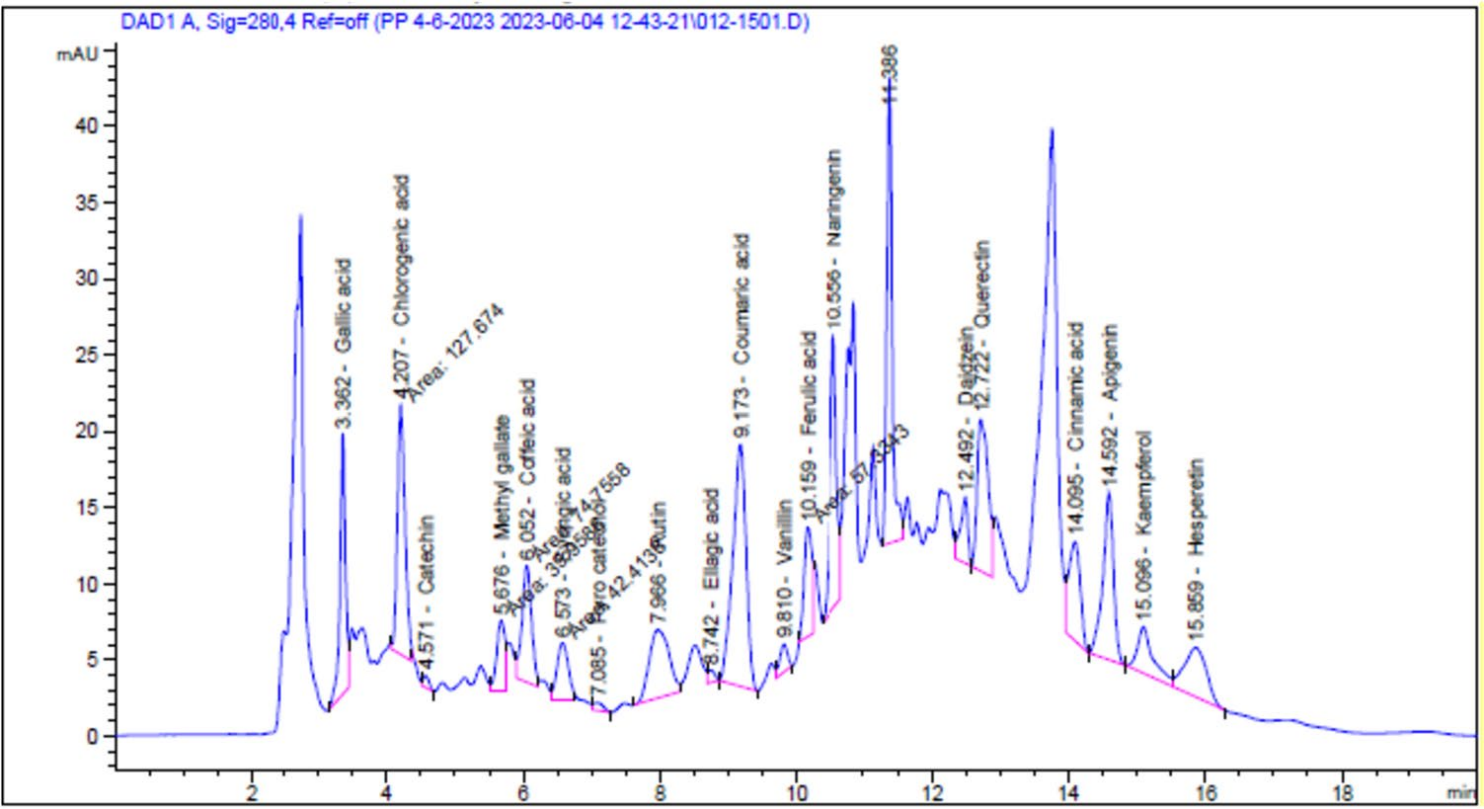
HPLC spectrum imprint profile of EchEE. 19 phenolic and flavonoid compounds were identified while peaks of the other constituents were not identified

The in vitro determination revealed that EchEE contains 18.51 ± 0.42 mg/g phenolic compounds and could scavenge 73 ± 3.11% of DPPH^•^ radical; also, the extract exhibited a valuable reducing power (Fig. 1). These data emphasized the potent antioxidant properties of this extract. In addition, the results of HPLC analysis revealed the identification of 19 phenolic and flavonoid compounds in the extract. From these ingredients, chlorogenic acid, naringenin, gallic acid coumaric acid, and caffeic acid were the major phenolic components while querectin, rutin, and apigenin were the major flavonoid components present in the extract (Fig. 2 and Table 1).

**Table 1:** Phenolic and flavonoid composition of EchEE identified by HPLC

	Area	Conc. (µg/ml = 20mg/ml)	Conc. (µg/g)
Chlorogenic acid	127.67	17.48	874.19
Naringenin	124.86	15.06	752.83
Gallic acid	94.15	8.13	406.51
Coumaric acid	214.12	6.76	337.75
Coffeic acid	74.76	5.75	287.44
Catechin	4.91	1.21	60.74
Methyl gallate	39.96	2.18	109.05
Syringic acid	42.41	2.88	143.80
Pyro catechol	5.23	0.75	37.62
Rutin	89.65	10.41	520.50
Ellagic acid	5.46	1.01	50.63
Vanillin	14.68	0.64	32.13
Ferulic acid	57.33	3.92	195.86
Daidzein	33.05	2.04	102.08
Querectin	111.91	15.42	770.75
Cinnamic acid	76.35	1.41	70.33
Apigenin	118.19	9.00	449.84
Kaempferol	48.40	3.75	187.65
Hesperetin	72.99	4.25	212.75

### Behavioral findings

**Fig. 3:**
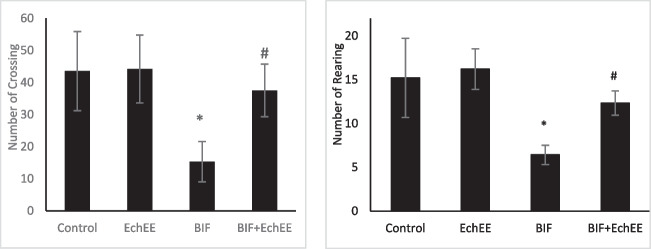
Open field test (number of crossings and rearing) behavior of BIF or/and EchEE treated rats. Data are represented as mean ± SD. * represents comparison with control, while # represents comparison with BIF group at *p* ≤ 0.05. EchEE is *Echinacea purpurea* ethanolic extract, BIF is Bifenthrin

The exploratory behaviors and motor activity of rats were assessed using an open field test. A significant decrease in number of squares crossings and rearing was observed in rats exposed to BIF compared with that of control ones (Fig. 3). Both locomotion and rearing significantly improved in the rats that were co-treated with EchEE together with BIF intoxication.

**Fig. 4:**
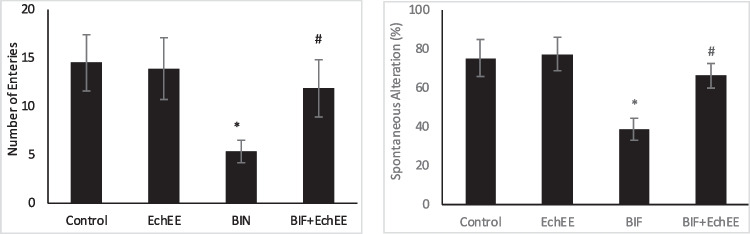
Y-Maze test (Number of arm entries and Spontaneous alternation percentage) of BIF or/and EchEE treated rats. Data are represented as mean ± SD. * represents comparison with control, while # represents comparison with BIF group at *p* ≤ 0.05. EchEE is *Echinacea purpurea* ethanolic extract, BIF is Bifenthrin

The short-term memory and locomotor activity were tested using Y-Maze test. The number of arm entries and the percentage of spontaneous alternation decreased significantly in BIF -treated group compared to that of the control ones, while the rats that received EchEE together with BIF showed a significant increase in the number of arm entries and spontaneous alternation percentage when compared with BIF-treated group (Fig. 4).

**Fig. 5:**
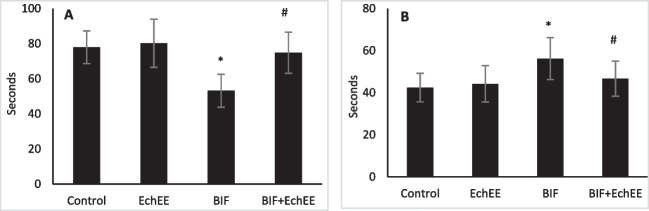
Modified elevated plus maze test, transfer latency-1 (**A**) and latency-2 (**B**) of BIF or/and EchEE treated rats. Data are represented as mean ± SD. * represents comparison with control, while # represents comparison with BIF group at *p* ≤ 0.05. EchEE is *Echinacea purpurea* ethanolic extract, BIF is Bifenthrin

A Modified elevated plus maze test showed that BIF-intoxication caused a significant increase in the level of anxiety-related behavior of rats as the values of transfer latency-1 (time spent in the open arms) decreased significantly, while the values of transfer latency-2 (time spent in the closed arms) increased significantly than those of controls (Fig. 5). Interestingly, the obtained data showed that co-treatment of rats with EchEE in line with BIF intoxication markedly restored the values of transfer latencies (1&2) toward those of the control group, indicating the anxiolytic effect of EchEE.

**Fig. 6:**
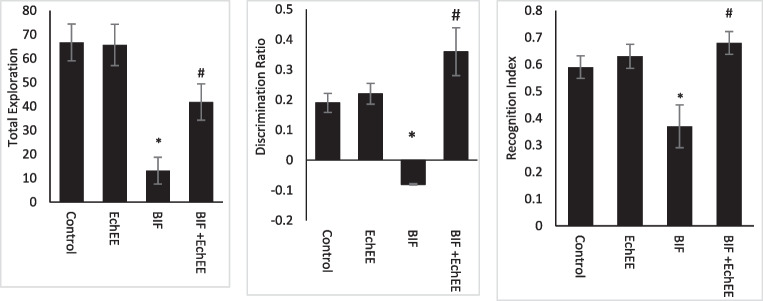
Novel object recognition (Total exploration, Discrimination ratio and Recognition Index) of BIF or/and EchEE treated rats. Data are represented as mean ± SD. * represents comparison with control, while # represents comparison with BIF group at *p* ≤ 0.05. EchEE is *Echinacea purpurea* ethanolic extract, BIF is Bifenthrin

Novel object recognition test declared that the rats treated with BIF exhibited impaired memory as the total exploration, discrimination rations and recognition index were reduced significantly compared to that of the controls. A significant improvement in these tests was observed in rats co-treated with EchEE besides BIF (Fig. 6). On the other hand, administration of EchEE alone did not negatively affect all the behavioral tests.

### Neurobiochemical findings

**Fig. 7:**
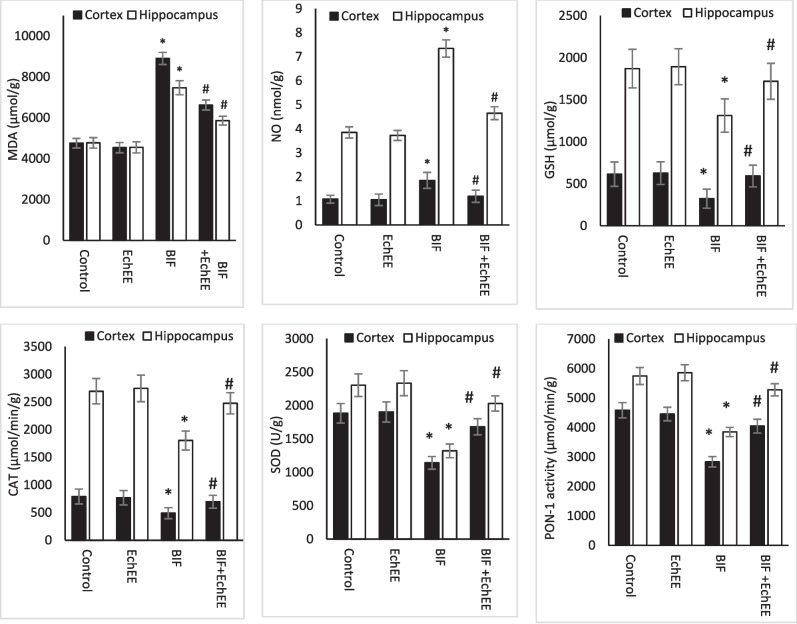
Oxidative stress status in brain regions of BIF or/and EchEE treated rats. Data are represented as mean ± SD. * represents comparison with control, while # represents comparison with BIF group at *p* ≤ 0.05. EchEE is *Echinacea purpurea* ethanolic extract, BIF is Bifenthrin

Oxidative stress status of the studied brain regions is illustrated in Fig. (7). The results of MDA and NO in the brain cortex and hippocampus showed a significant increase in the animals exposed to BIF, while the values of the antioxidants (CAT, SOD, PON-1 and GSH) in both brain areas were significantly decreased compared with their values in the controls. On the other hand, insignificant difference was recorded in oxidative stress markers in both brain areas of the animals treated with the EchEE alone; this indicates the neuro-safety of this extract. In a promising manner, animals co-treated with EchEE together with BIF showed a significant improvement in the values of the above-mentioned oxidative stress markers when compared with corresponding values of the BIF group.

**Fig. 8:**
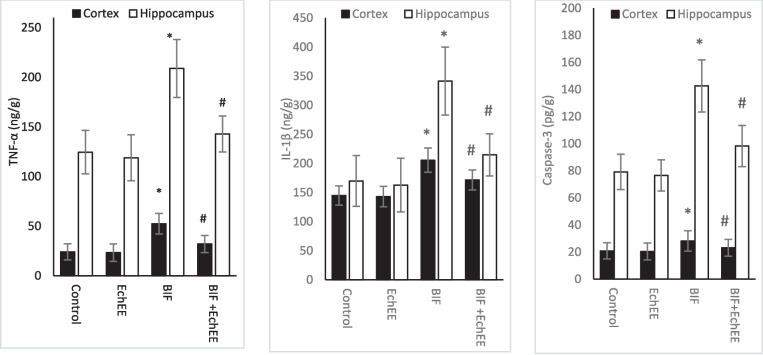
Inflammatory cytokines level and caspase-3 in brain regions of BIF or/and EchEE treated rats. Data are represented as mean ± SD. * represents comparison with control, while # represents comparison with BIF group at *p* ≤ 0.05. EchEE is *Echinacea purpurea* ethanolic extract, BIF is Bifenthrin

In Fig. (8), the animals treated with BIF exhibited a significant rise in the apoptotic marker (caspas-3) and the pro-inflammatory cytokines (TNF and IL-1ß) of cortex and hippocampus. While as no disturbance was observed in these markers in the animals that received EchEE alone in compared to the control group. Interestingly, co-treatment of rats with EchEE together with BIF intoxication resulted in a significant improvement in the levels of caspas-3, TNF and IL-1ß in both brain areas, although it was still higher than in the control values.

**Fig. 9:**
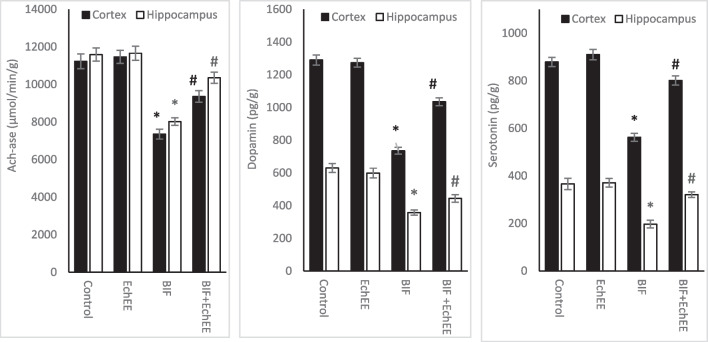
Acetylcholinesterase (AChE-ase) activity and biogenic amines level in brain regions of BIF or/and EchEE treated rats. Data are represented as mean ± SD. * represents comparison with control, while # represents comparison with BIF group at *p* ≤ 0.05. EchEE is *Echinacea purpurea* ethanolic extract, BIF is Bifenthrin

Comparing with the control group, BIF was found to markedly inhibited AChE-ase activity, and significantly decreased dopamine and serotonin levels in both cortex and hippocampus, while treatment with EchEE alone did not produce any unfavorable effects on the values of the mentioned parameters. Favorably, co-treatment of animals with EchEE together with BIF-intoxication was found to be effective in restoring the values of AChE-ase, dopamine and serotonin in both brain areas (Fig. 9).

## Discussion

Neurotoxicity can be identified as any disturbing effect on either central or peripheral nervous system caused by physical, biological or/and chemical factors **(**Costa et al. 2008**)**. This study was carried out to evaluate the chemo-modulatory effect of the ethanolic extract of *Echinacea purpurea* flowers against neurotoxicity, neurobehavioral and neuro-inflammatory deteriorations induced by bifenthrin (BIF) in animals. BIF is metabolized, mainly by cytochrome P450 in the liver **(**Nieradko-Iwanicka et al. 2015**),** and led to generation of reactive oxygen species (ROS) that play a key role in the oxidative stress and cause an oxidative damage in the organism **(**Sadowska-Woda et al. 2010; El-Demerdash 2011**)**. Due to lipophilic property of pyrethrinoids, it can also accumulate in cell membranes and deactivate them **(**Ogaly et al. 2015**)**. The high rate of oxidative metabolism and the great amounts of polyunsaturated fatty acids make the brain vulnerable to oxidative damage by ROS **(**Dua and Gill 2001; Sharma et al. 2007**)**.

In the current study, the levels of MDA and nitric oxide (NO) in both cortex and hippocampus were significantly increased, while the values of the antioxidants (CAT, SOD, PON-1 and GSH) were significantly decreased in BIF -treated group compared with their values in the control group. These results are confirmed by previous studies that reported the toxicity of BIF on brain tissue **(**Gargouri et al. 2018b; Gregory et al. 2018; Syed et al. 2018**)**. Elevated levels of MDA and NO, in these areas, indicate increased peroxidation of cell membrane and consequently the presence of oxidative stress, which leads to loss of membrane structure and function **(**Syed et al. 2018**)**. Numerous reports have indicated a close correlation between oxidative stress and neurodegeneration **(**Wang et al. 2014; Kim et al. 2015; Gargouri et al. 2017**)**.

The obtained results clearly indicated also that the antioxidants CAT, SOD, GSH play a role in the elimination of free radical by their scavenging or stabilizing effects **(**Anandakumar et al. 2013**)**. Free radicals attack the unsaturated fatty acids in the cell membrane; consequently, stimulate and support lipid peroxidation, which is a key process in many disease states, and considered one of the reactions caused by oxidative stress **(**Schinella et al. 2002**)**. Decreasing CAT and SOD activities reinforces generation of hydrogen peroxide and superoxide radicals in the brain; further, this leads to peroxidation of membrane lipid, resulting in increased MDA level **(**Syed et al. 2018**)**. PON-1, an enzyme synthesized in the liver **(**Leviev et al. 1997**),** has antioxidant and anti-inflammatory ability **(**Aviram et al. 1998; Aviram and Vaya 2013**),** and can detoxify xenobiotics, such as organophosphates **(**Costa et al. 1999, 2013**)**. Salazar et al. (2021) suggested that PON-1, carried on high-density lipoproteins, can cross the blood–brain barrier, and transport to brain cells where it can prevent the oxidative stress and lipid peroxidation in certain types of brain diseases.

In the current study, the intake of EchEE alone had no unfavorable effects on all tested parameters, indicating the safety of this extract. Co-administration of EchEE besides BIF-intoxication resulted in a significant improvement in the estimated oxidative stress markers. The inhibition of lipid peroxidation (MDA) and NO formation, coupled with the prevention of GSH exhaustion and reactivation of the endogenous antioxidant enzymes (CAT, SOD and PON-1) by EchEE may be attributed to its free radical scavenging and stabilizing potential of its constituents. The phytochemical analysis of the EchEE by HPLC revealed the identification of 19 flavonoids and phenolic compounds including chlorogenic acid, naringenin, gallic acid, syringic acid, ferulic acid, vanillin, caffeic acid, coumaric acid, querectin. These compounds are powerful antioxidants that directly work to scavenge free radicals and prevent lipid peroxidation **(**Lee et al. 2003**).**

The results of the current study declared that BIF markedly increased TNF and IL-1ß levels in cortex and hippocampus. It was established that BIF elevates the levels of TNF and IL-1ß in hippocampus **(**Gargouri et al. 2018b**)**. TNF and IL-1ß are pro-inflammatory cytokines released post NF-kB activation during an inflammatory process **(**Liu et al. 2017; Gargouri et al. 2018b**)**. The microglial cells are the main immune cells in the brain and play an essential role in the regulation of neuroinflammation and amyloid-B deposition **(**Villegas-Llerena et al. 2016**)**. Over activity of microglial cells leads to over-release of pro-inflammatory cytokines, such as TNF and IL-1β, which are also reported to promote amyloid protein expression and amyloid-B formation **(**Lee et al. 2015**)**. Increased levels of these cytokines induce the activation of cyclooxygenase-2 (COX-2) and inducible nitric oxide synthase (iNOS) **(**Gabay et al. 2010**)** which is responsible for the production of NO that, in its turn, plays a key role in the mediation of oxidative stress, when it is released in high amounts **(**Mani et al. 2018**)**.

The current study declared that the level of Caspas-3 displayed a significant enhancement in both brain areas in BIF –treated rats. Gargouri et al. (2019) documented that administration of BIF to adult rat results in apoptosis as evidenced by a significant rise in the level of the apoptotic marker, Caspas-3, in hippocampus. The pro-apoptotic caspase cascade was accelerated by the increased release of TNF and IL-1β during the inflammatory process **(**Tacke et al. 2009**)**. Additionally, it was suggested by Yu et al. (2008) that the oxidative stress induced by the pesticides is the common mechanism that leads to DNA fragmentation and apoptosis.

The present data revealed that EchEE can downregulate the elevated levels of TNF, IL-1ß and Caspas-3 in both cortex and hippocampus of the rats co-treated with EchEE besides BIF. EchEE has been found to restrain the release of TNF-α and IL-1ß, this was attributed to its anti-inflammatory property **(**Hájos et al. 2012; Hudson 2012). The identified gallic acid that is found in a high concentration in the EchEE has been shown to inhibit the release of inflammatory cytokines by microglia **(**Dong et al. 2022**).** Chicoric acid, the major polyphenolic compound present in EchEE, which may be present in our extract but not identified, and also quercetin and p-coumaric acid downregulated LPS-induced glial activation by inhibiting the pathways of NF-kB and MAPK **(**Liu et al. 2017; Han et al. 2021; Daroi et al. 2022**).**

Also, Zheng et al. (2022) reported the protective effect of chlorogenic acid, a major phenolic compound present in EchEE, against hypoxic-ischemic brain injury by reducing inflammation and oxidative stress via regulating the Nrf2-NF-κB signaling pathway. Chlorogenic acid was found to mitigate also the elevated levels of caspase-3 and caspase-7 expression in focal cerebral ischemic rats (Shah et al. 2021**)**. In addition, the study of Poland et al. (2009) indicated that the 3,4,5-trihydroxycinnamic acid, a caffeic acid derivative isolated from Echinacea extract, can inhibit BV2 microglial cells inflammation via inhibition of iNOS.

Moreover, the obtained data showed that acetylcholinesterase (AChE) activity decreased significantly in the cortex and hippocampus of BIF –treated rats; this result is in line with the results of Syed et al. (2018), Gargouri et al. (2019) and Gomaa et al. (2021) who stated that BIF can decrease AChE activity in the brains of exposed animals. AChE hydrolyzes acetylcholine, an essential neurotransmitter, to generate choline and acetate in the synaptic cleft **(**Soreq 2015**)**; inhibition of AChE activity is associated with the increase in lipid peroxidation, which decreases cellular metabolism, induces distortion of cell membrane, and disrupts the metabolic and nervous activities as a consequence **(**Suresh et al. 1992**)**.

Also, dopamine and serotonin levels recorded a significant decrease in both cortex and hippocampus of BIF –intoxicated rats; these results reflect that BIF affects brain function by disturbing the dopamine, cholinergic and serotonin systems **(**Ansari et al. 2012a, b; Syed et al. 2018**)**. Pyrethroids have been reported to affect the release of the neurotransmitters via reducing chloride currents through voltage-dependent chloride channels **(**Soderlund et al. 2002**),** i.e. inhibiting chloride influx **(**Bloomqiust et al. 1986**)**, which is responsible for neurological effects **(**Tayebati et al. 2009**)**.

On the other hand, animals co-treated with EchEE besides to BIF showed a significant increase in AChE activity, as well as dopamine and serotonin levels, in both cortex and hippocampus in compared to BIF –treated rats. Velíšek (2006) stated that Echinacea extract counteracted dopamine, acetylcholine, opioids, and the GABAergic system in the brain; interestingly, Konishi et al. (2005)**, **Woelkart et al. (2009) and Baldin et al. (2022) attributed this effect to the active components inside the extract such as cafeic acid, rosmarinic acid, and gallic acid that can easily pass through the blood–brain barrier and exert their protective effect. Also, results of Zhang et al. (2023) revealed that the naringenin and apigenin treatment counteracted corticosterone-induced depressive behaviors by elevating the levels of 5- serotonin, dopamine and norepinephrine in the hippocampus. In addition, the study of Wang et al. (2022) indicated that chicoric acid can prevent dopaminergic lesions, motor deficits and glial activation in mice with Parkinson disease, and increase neurotrophic factor, dopamine, and serotonin levels in brain striatum.

Behavior is the result of various motor, sensory and associative functions of the nervous system; the assumption is that insecticides negatively affect one or more of these functions by disrupting memory and learning processes, and cause adverse behavioral effects **(**Gargouri et al. 2018a**)**. The results of behavioral tests of the current study revealed that BIF enhanced anxiety level in rats, as manifested by elevated plus-maze test. Similar effect has been reported by Gregory et al. (2018) who stated that BIF induce neuronal changes in the striatum and cortex of rats which may be related to neuroinflammation and oxidative damage through activation of Nrf2/NF-kBp65 pathway that may enhance anxiety-like behavior.

Co-administration of EchEE together with BIF significantly decreased anxiety level; the anxiolytic ability of *Echinacea* preparations has been recognized previously in experimental animals **(**Haller et al. 2010; Hájos et al. 2012**)**. It was reported that *Echinacea purpurea* contains components, like gallic acid which can easily cross the blood–brain barrier and affect the brain centers related with anxiety **(**Jafaripour et al. 2022**).** Also, this plant contains chlorogenic acid, which was found able to decrease anxiety in laboratory animals **(**Bouayed et al. 2007**).** Additionally, Takeda et al. (2003) suggested that the caffeic acid, a major component of *Echinacea purpurea* extract, markedly decreased anxiety through indirect modulation of alpha1A- adrenoceptor system in the brain.

Also, the behavioral tests revealed that rats received this insecticide exhibited exploratory behavioral and spatial learning impairments, as well as memory and locomotion dysfunction. These obtained results are in line with the findings of several researchers who found that dopaminergic and serotonergic neurodegeneration correlate with locomotion, learning, and memory impairment **(**Ansari et al. 2012a, b; Gargouri et al. 2018a**; **Syed et al. 2018**)**. Other researchers have stated that learning, memory, and emotional defects are mediated also by inhibition of the AChE enzyme **(**Syed et al. 2018**)**. Moreover, it was suggested that oxidative damage in the hippocampus and cholinergic dysfunction after BIF-exposure could be involved in cognitive function, memory, and learning deficits **(**Gargouri et al. 2017, 2018a**)**. Cytokines, including TNF, IL-1 ß, and IL-6, have been described to affect the synaptic plasticity and neurogenesis which are implicated in learning and cognitive impairment **(**McAfoose and Baune 2009; Terrando et al. 2010).

Co-administration of EchEE besides BIF intoxication markedly reduced the exploratory and spatial learning impairment, and improved memory and locomotion loss. Liu et al. (2017) have concluded that chicoric acid improved learning and memory impairment in lipopolysaccharide-induced learning and memory loss in mouse; they suggested that this component exerts its improving-effect via suppressing NF-kB transcriptional pathway. In recent studies, chlorogenic acid and p-Coumaric acid were found to counteract the impairment of learning and cognitive ability and maintain the long-term spatial memory in experimental animals (Zheng et al. 2022; Daroi et al. 2022).

Finally, the mechanism that involved in the neuroprotective effect of EchEE based mainly on its powerful antioxidant activity that appeared from the data of DPPH^•^ radical scavenging activity and reducing power ability. The antioxidant activity of EchEE may be attributed mainly to its phenolic and flavonoid contents. These compounds prevent free radicals from attacking macromolecules such as DNA, proteins and lipids; and thus prevent inflammation, peroxidation of membrane lipids and exhaustion of the endogenous defensive capacity. This in turn stabilizes the membrane permeability and integrity. This leads to improvement of brain function demonstrated by restoring AChE, dopamine and serotonin values that decreased by BIF and consequently improvement of the behavioral activity.

In conclusion, the obtained results demonstrated that co-administration of EchEE in line with BIF intoxication can preserve and regenerate the integrity of the neural membranes and prevent neural damage through preventing the oxidative stress. This leads to amelioration of the neurotransmitter release and improvement of behavioral activity as a consequence; this beneficial effect may be attributed to the high antioxidant component in the extract.

## Data Availability

All data generated or analyzed during this study are included in this published article.
